# Stimulation of Human Adenovirus Infection Modulated by Emerging Micropollutants

**DOI:** 10.1007/s12560-025-09676-w

**Published:** 2026-01-22

**Authors:** Catielen Paula Pavi, Yasmin Ferreira Souza Hoffmann Jempierre, Lucas Zanchetta, Paula Rogovski, Gislaine Fongaro

**Affiliations:** Laboratory of Applied Virology, Department of Microbiology, Immunology and Parasitology, Federal University of Santa Catarina, Campus Universitário Trindade, Florianópolis, Santa Catarina 88040-900 Brazil

**Keywords:** Environmental virology, Micropollutants, Antibiotics, Microplastics, Viral replication

## Abstract

Contaminants of emergent concern (CECs), such as pharmaceuticals and microplastics, are increasingly found in aquatic environments, yet their interactions with viral pathogens remain underexplored. This study evaluated the effects of antibiotics, antidepressants, microfibers, and nanoplastics on human adenovirus type 5 (HAdV-5) replication in A549 cells. A series of in vitro assays simulating distinct exposure scenarios across the viral replication cycle was conducted. Results showed that individual pre- or post-infection exposure to CECs did not significantly impact HAdV-5 replication. However, co-incubation of CECs with viral particles at physiological temperature (37 °C) led to a significant increase in viral replication up to 1.5 log₁₀ compared to viral control, highlighting temperature-dependent interactions. No enhancement was observed at room temperature. The findings suggest that CECs can modulate viral infectivity through direct physicochemical interactions, particularly under conditions resembling those of wastewater environments. This study provides new insights into the potential risks posed by the co-occurrence of viruses and CECs in aquatic ecosystems.

## Introduction

In recent decades, the widespread detection of micropollutants in aquatic environments has raised growing concerns regarding their ecological and human health impacts. These substances, ranging from pharmaceuticals and personal care products to micro and nanoplastics (MNPs), are continuously released into surface waters through domestic, hospital, and industrial wastewater, often without complete removal by conventional treatment systems (Tumwesigye et al., 2023). Due to their chemical persistence and biological activity, even low concentrations of these compounds can induce subtle but significant biological effects in exposed organisms (Moghadam et al., 2023).

Among the various classes of micropollutants, pharmaceutical residues such as carbamazepine, ciprofloxacin, sulfamethoxazole, and trimethoprim are of particular interest because of their high detection frequency and potential to promote antimicrobial resistance (Gupta et al., 2024). Similarly, MPs have gained attention not only for their physical accumulation but also for their capacity to act as carriers of a wide range of environmental contaminants, including persistent organic pollutants, pharmaceuticals such as antibiotics and neuroactive drugs, pesticides, and endocrine-disrupting chemicals, that readily adsorb to their surfaces (Fu et al., 2021). Once internalized by organisms, these contaminant-loaded particles can interfere with cellular processes, such as inducing oxidative stress through ROS overproduction, disrupting membrane integrity, impairing protein processing, and altering key physiological functions including photosynthesis in algae and metabolic homeostasis in animal cells. The complex physicochemical interactions between MNPs, their adsorbed pollutants, and biological systems highlight the importance of comprehensive toxicity assessments that consider both particle-specific effects and the enhanced toxicity arising from co-exposure to associated chemicals.

Despite the recognized importance of viruses in aquatic environments, the potential interactions between viruses and micropollutants remain largely unexplored. Emerging evidence suggests that chemical contaminants such as pharmaceuticals and microplastics (MP) could influence viral stability, infectivity, and replication, either by direct physicochemical interactions or indirectly by altering host cell responses (Li et al., 2024). Understanding these interactions is crucial, as they may affect virus persistence and transmission, ultimately impacting public health and environmental safety.

Viruses are ubiquitous in the environment and play critical roles in ecosystem dynamics as well as public health. Among them, human adenoviruses are frequently detected in water bodies contaminated with sewage effluents and are considered important indicators of viral pollution due to their resistance to environmental degradation and disinfection processes (Rachmadi et al., 2020). Adenoviruses can cause a range of diseases, from mild respiratory infections to more severe illnesses in immunocompromised individuals, making their monitoring in environmental matrices essential for assessing viral contamination risks (Suraka et al., 2022). HAdV-5 was specifically chosen for this study because it is one of the most environmentally persistent adenovirus types and is also one of the most extensively characterized viral models in vitro. Its well-established replication kinetics, robust growth in A549 cells, and wide use in virology studies strengthen its suitability for controlled mechanistic evaluations, which aligns with the in vitro design of the present work.

The primary objective of this study was to evaluate the interactions between selected micropollutants and human adenovirus during in vitro cultivation. We specifically investigated whether exposure to environmentally relevant concentrations of micropollutants alters adenovirus replication dynamics in cultured cells. This work aims to provide novel insights into how chemical contaminants may modulate viral behavior, contributing to a more comprehensive understanding of environmental virology and pollutant impacts.

## Methodology

### Cells and Viruses

In vitro assays were conducted using the human lung adenocarcinoma cell line A549 (ATCCCCL-185), cultured in Minimum Essential Medium (MEM) supplemented with fetal bovine serum (FBS), under standard conditions (37 °C, 5% CO₂, humidified atmosphere). The viral model used was human adenovirus type 5 (HAdV-5), with viral stocks prepared according to Simões et al. (1999) and titrated via the TCID₅₀ method. Viral suspensions were stored at − 80 °C until use.

### Micropollutants

The selected CECs represented a range of micropollutants commonly detected in wastewater and aquatic environments. These included antibiotics (ciprofloxacin, trimethoprim, sulfamethoxazole, and amoxicillin), neuroactive pharmaceuticals (fluoxetine and sertraline), and carbamazepine. All pharmaceuticals were originally purchased from Sigma-Aldrich and kindly donated for this study by the Resource Recovery in Sanitation Systems Study Group (RReSSa/UFSC). In addition, two types of MNPs were included: polyester microfibers (classified as microplastics, ranging from 44.7 μm to 911.0 μm) (Lins et al., 2024) and polystyrene/aluminum oxide nanoparticle suspensions (classified as nanoplastics, average diameter 90.1 ± 4.9 nm) (Vicentini et al., 2019). These MNPs were produced in-house and provided by the Laboratory of Aquatic Contamination Biomarkers and Immunochemistry (LABCAI/UFSC). All CECs used in this study were obtained through collaborations with UFSC research groups, reinforcing the methodological reliability and traceability of the materials employed.

### Analytical Validations

#### Cytotoxicity Assays

Cytotoxicity of the pharmaceutical compounds was evaluated via the sulforhodamine B (SRB) colorimetric assay (Vichai & Kirtikara, 2006). A549 cells were exposed to compound concentrations (Table 1) based on literature and human serum levels, followed by 72 h incubation. Fixation was performed with trichloroacetic acid, SRB staining applied, and absorbance read at 510 nm. CC₅₀ values were calculated according to the analytical approach outlined by Vichai & Kirtikara, using dose–response curves generated from the normalized absorbance data.

**Table 1:** Concentration range of pharmaceutical-class micropollutants tested using the SRB assay

Pharmaceutical	Concentration range (µg/mL)	Reference
Carbamazepine	5 to 0.005	Grześk et al., 2021
Ciprofloxacin	3 to 0.003	Vella et al., 2015
Fluoxetin	2 to 0.002	Lundmark et al., 2001
Sertralin	2 to 0.002	Huddart et al., 2020
Trimetoprim	2 to 0.002	Dudley et al., 1984
Sulfamethoxazole	60 to 0.06	Dudley et al., 1984
Amoxicilin	50 to 0.05	Fonseca et al., 2003

For MNPs, a customized environmental cytotoxicity assay previously developed by our group was employed (Rigotto et al., 2005). Cells were exposed to three concentrations of MNPs for 1 h, followed by medium replacement and 72 h incubation. Cell viability was assessed via naphthol black staining.

#### Methodological Validation of RT-qPCR

To ensure the reliability of RT-qPCR quantification in the presence of micropollutants, a methodological validation assay was performed. Based on the cytotoxicity results, four different concentrations were selected for each micropollutant of interest (Table 2). These concentrations represented subcytotoxic levels and were within the range commonly detected in environmental and biological matrices.

**Table 2:** Range of micropollutant concentrations (µg/mL) employed in the methodological validation of RT-qPCR

Micropollutant	Test 1	Test 2	Test 3	Test 4
Carbamazepine	1	0.25	0.0625	0.015625
Ciprofloxacin	1	0.25	0.0625	0.015625
Fluoxetin	1	0.25	0.0625	0.015625
Sertralin	1	0.25	0.0625	0.015625
Trimetoprim	1	0.25	0.0625	0.015625
Sulfamethoxazole	5	1.25	0.03125	0.078125
Amoxicilin	5	1.25	0.03125	0.078125
Microplastic	5	1.25	0.03125	0.078125
Nanoplastic	5	1.25	0.03125	0.078125

Each micropollutant was directly added to a suspension containing the purified genetic material of HAdV-5, in the absence of host cells. The resulting mixture was then transferred to an RT-qPCR reaction plate prepared with the appropriate master mix and primers targeting HAdV-5, in accordance with Sect. 2.5. An RT-qPCR kit was used to maintain the same conditions applied for mRNA quantification, without DNase I treatment. The cycling conditions followed the protocol established for viral quantification in this study (session 2.5).

This assay aimed to evaluate whether the selected micropollutants interfered with the amplification efficiency or detection of the viral genome, either by inhibiting enzymatic reactions, altering fluorescence signals, or interacting with nucleic acids. The results from this control experiment were used to validate the absence of analytical artifacts and to support the interpretation of downstream experiments involving viral replication in cell culture.

### HAdV-5 Replication Under Micropollutant Exposure

**Fig. 1:**
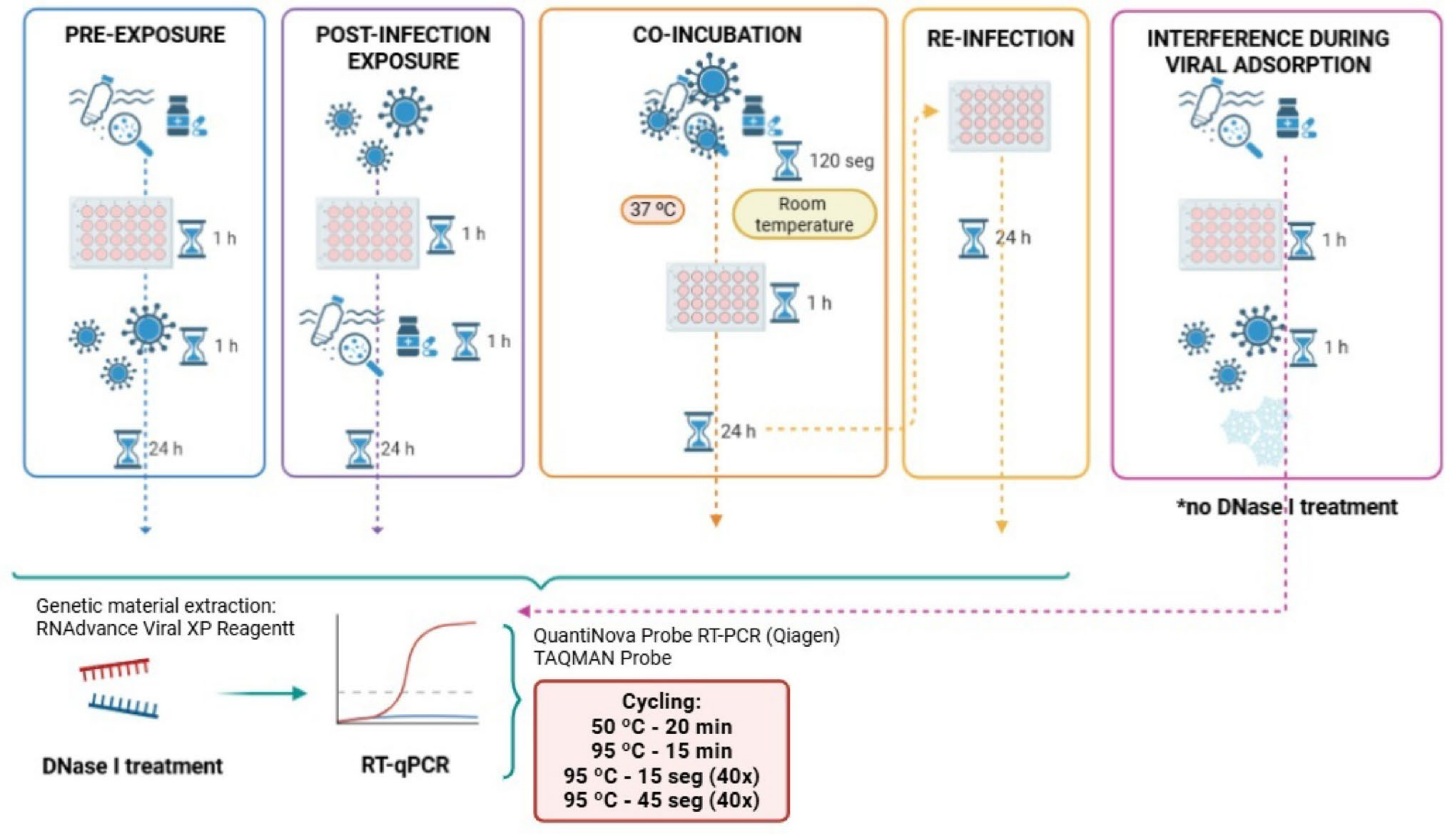
Experimental scenarios designed to assess the effects of CECs exposure on HAdV-5 replication

To investigate the impact of CECs on HAdV-5 replication, a series of in vitro assays were originally developed for this study, rather than adapted from previously published protocols, in order to simulate distinct exposure scenarios across the viral replication cycle (Fig. 1). The A549 cell line was used throughout, and all infections were performed with HAdV-5 at a concentration of 10^4^ TCID₅₀/mL, with incubation at 37 °C and 5% CO₂ in a humidified atmosphere. The micropollutants were tested at environmentally relevant and biologically tolerable concentrations (Table 3). All essays included viral control (positive control, with HAdV-5 suspension only) and a cell control (negative control, cells with medium only).

**Table 3:** Concentrations of micropollutants used across all assays

Micropollutant	Concentration on the assays (µg/mL)
Carbamazepine	2
Ciprofloxacin	2
Fluoxetin	2
Sertralin	2
Trimetoprim	2
Sulfamethoxazole	10
Amoxicilin	10
Microplastic	10
Nanoplastic	10

#### Pre-Exposure of Cells to Micropollutants

This assay aimed to evaluate whether prior contact of CECs with host cells could modulate susceptibility to viral infection. A549 cells were exposed to CECs for 1 h at 37 °C. After this incubation, the medium containing CECs was carefully aspirated, and the cells were washed with phosphate-buffered saline (PBS) to remove any residual compounds. Subsequently, cells were infected with HAdV-5 and incubated for 1 h to allow virus adsorption. After infection, the viral inoculum was removed, and fresh MEM supplemented with FBS was added. Cells were incubated for 24 h to allow viral replication before being harvested for downstream analysis.

#### Post-Infection Exposure to Micropollutants

In this protocol, designed to assess whether CECs interferes with viral replication after successful entry, cells were first infected with HAdV-5 for 1 h. Following adsorption, the viral suspension was aspirated and replaced with medium containing the test MP. After 1 h of exposure, fresh medium (without removing the MP) was added, and the cells were incubated for an additional 24 h. This allowed evaluation of CECs effects on intracellular stages of viral replication and progeny virus production.

#### Interference During Viral Adsorption

To investigate whether CECs interfere directly with the virus-cell binding step, a pre-exposure of A549 cells to CECs was carried out for 1 h, followed by infection with HAdV-5 for 1 h. Immediately after infection, plates were transferred to 4 °C for 15 min to inhibit endocytosis and stabilize the adsorption phase. This temperature shift selectively halts virus entry by increasing membrane rigidity and preventing fusion, while allowing viral adsorption to proceed (Aboud, Shoor, Salzberg; 1979; White et al., 2023), enabling assessment of CECs effects on early attachment processes. Cells were then harvested for nucleic acid analysis.

#### Virus-Micropollutant Co-Incubation

This assay assessed whether CECs interacts directly with viral particles, potentially altering infectivity. HAdV-5 was mixed with each CEC at a 1:1 ratio (50 µL of HAdV-5 suspension and 50 µL of the CEC solution, both diluted in MEM) and incubated for 2 min at room temperature; an identical mixture was incubated at 37 °C to assess potential temperature-dependent differences. The mixture was then diluted in culture medium containing 2% FBS and applied to A549 cells. After 1 h, the inoculum was removed, and fresh medium was added. Cells were incubated for 24 h to allow replication of any virus that retained infectivity. This assay mimics environmental or physiological scenarios of simultaneous exposure.

#### Progeny Virus Reinfection Assay

To determine the biological relevance of changes in viral replication, an in vitro reinfection assay was performed. Supernatants from the co-exposure assay were collected after 24 h and transferred to fresh A549 monolayers, which were then incubated for an additional 24 h. Viral load was quantified as HAdV-5 mRNA, ensuring that only actively replicating virus was measured. This approach allowed us to assess whether prior exposure to CECs resulted in increased or decreased production of infectious progeny, providing insight into longer-term effects on viral fitness. Each experimental condition was compared against its respective viral and cell-only controls. After incubation, samples were processed for RNA extraction and ICC-RT-qPCR quantification of HAdV-5 mRNA, as described below.

### Nucleic Acid Extraction and RT-qPCR

Adenoviral replication was quantified through integrated cell culture coupled with reverse-transcription quantitative PCR (ICC-RT-qPCR), following the approach described by Fongaro et al. (2013). This method ensures that only actively replicating virus is detected, since quantification is based on viral mRNA transcribed during infection, rather than on residual input viral DNA.

After cell infection and incubation, nucleic acids were extracted using the RNAdvance Viral XP kit (Beckman Coulter). To eliminate any remaining adenoviral genomic DNA, all extracts were treated with DNase I (1 U; Invitrogen) prior to amplification. This step prevents background amplification from contaminating DNA and guarantees that downstreamdetection reflects only RNA-derived products.

RT-qPCR was performed using the QuantiNova Probe RT-PCR Kit (Qiagen), which incorporates the reverse-transcription step within the qPCR reaction. Although the primers and probe target the conserved hexon gene, commonly used for adenoviral DNA detection, their use is also fully appropriate for mRNA quantification. This is because the hexon transcript is one of the most abundant and conserved early-to-late mRNAs produced during adenovirus infection, and its sequence is identical in both RNA and DNA templates. Consequently, once genomic DNA is removed by DNase treatment, hexon-specific oligonucleotides reliably amplify only viral mRNA, serving as a robust transcriptional marker of active replication. Cycling conditions and oligonucleotide sequences used in this assay are detailed in Tables 4 and 5.

**Table 4:** RT-qPCR setup: primers and probe for HAdV-5

Component	Sequence	Reference
Probe	FAM-CCGGGCTCAGGTACTCCGAGGCGTCCT-TAMRA	Hernroth et al. (2002)
Forward Primer (F)	CWTACATGCACATCKCSGG	
Reverse Primer (R)	CRCGGGCRAAYTGCACCAG	

**Table 5:** RT-qPCR setup: thermal cycling conditions

Step	Temperature	Time	Cycles
Reverse Transcription	50 °C	20 min	1
Initial Denaturation	95 °C	15 min	1
Denaturation (cycling)	95 °C	15 s	40
Annealing/Extension	60 °C	45 s	40

### Statistical Analysis

To ensure consistency across all experimental conditions, the same viral stock (a single production batch of HAdV-5) was used throughout the study. This stock was previously quantified by qPCR to determine the genome copy number and verify uniformity of the inoculum, resulting in an HAdV-5 input of 4.94 × 10⁴ GC/mL in each assay. In addition, infectious titers were independently assessed by TCID₅₀, which confirmed the same pattern of consistency across experiments, reinforcing the reliability of the viral input used in all assays.

For all ICC-PCR analyses, genome copy numbers obtained from duplicate cell populations were converted to log₁₀ values, and the mean of each duplicate was used for quantification. To allow direct comparison across independent experiments, data were normalized by setting the viral control of each experimental run as the reference point. The mean log₁₀ value of the viral control was therefore normalized to 1, representing the maximum replication observed for the baseline condition. Values greater than 1 indicate an increase in HAdV-5 replication relative to the viral control, whereas values below 1 reflect a reduction in replication under the different micropollutant exposure scenarios. This normalization approach ensured consistency across experiments and allowed relative changes in viral replication to be compared independently of the absolute viral titers.

Parametric analysis was performed using ANOVA followed by Dunnett’s post hoc test. Non-parametric tests (e.g., Kruskal-Wallis, Brown-Forsythe) were also employed to validate findings. Differences in HAdV-5 replication were considered statistically significant when *p* < 0.05. All experiments were performed in triplicates, with each sample analyzed in duplicate.

## Results

### Control Assays: Cytotoxicity and Methodological Validation of qPCR

To ensure robustness and accurate interpretation of the experimental results, two preliminary control assays were performed. First, the cytotoxicity of all selected micropollutants was assessed in A549 cells using the SRB assay to confirm that the concentrations applied did not compromise cell viability through nonspecific cellular damage. Second, a methodological control was conducted to evaluate potential interference of the micropollutants with the RT-qPCR assay itself. This step was critical to exclude any direct effects of the tested compounds on enzymatic amplification or fluorescence detection, thereby ensuring that observed variations in viral replication truly reflect biological phenomena rather than analytical artifacts.

Table 6 summarizes the CC_50_ values obtained from the SRB assay to the micropollutants classified as pharmaceuticals, which are consistent with the literature and informed the selection of concentrations for subsequent experiments. Unlike the tested pharmaceuticals, MNPs do not have a defined chemical structure and do not act as soluble bioactive agents. As solid particles or colloidal dispersions, their interactions with cells do not follow classic dose-dependent toxicity mechanisms typically observed with chemical compounds. Therefore, the calculation of the CC₅₀ (the concentration that reduces cell viability by 50%) is not suitable or applicable to these types of materials. Instead, cytotoxicity was assessed qualitatively based on cell viability observations at different concentrations (20, 10 and 5 µg/mL), without generating dose–response curves typically used for pharmacological substances. The non-cytotoxic concentration observed for CECs was 10 µg/mL, which was therefore used for the following assays.

**Table 6:** Results from SRB assay to micropollutants characterized as pharmaceuticals

Contaminant of Emerging Concern	CC₅₀ (µg/mL)	95% Confidence Interval (µg/mL)	
Carbamazepine	7.28		3.25–13.63
Ciprofloxacin	> 5		0.071–1000.00*
Fluoxetine	3.64		2.69–4.90
Sertraline	32.95		5.50–198.70
Trimethoprim	1.20		1.10–6.63
Amoxicillin	> 50		53.90–1000.00*

*Exceeds tested range

**Fig. 2:**
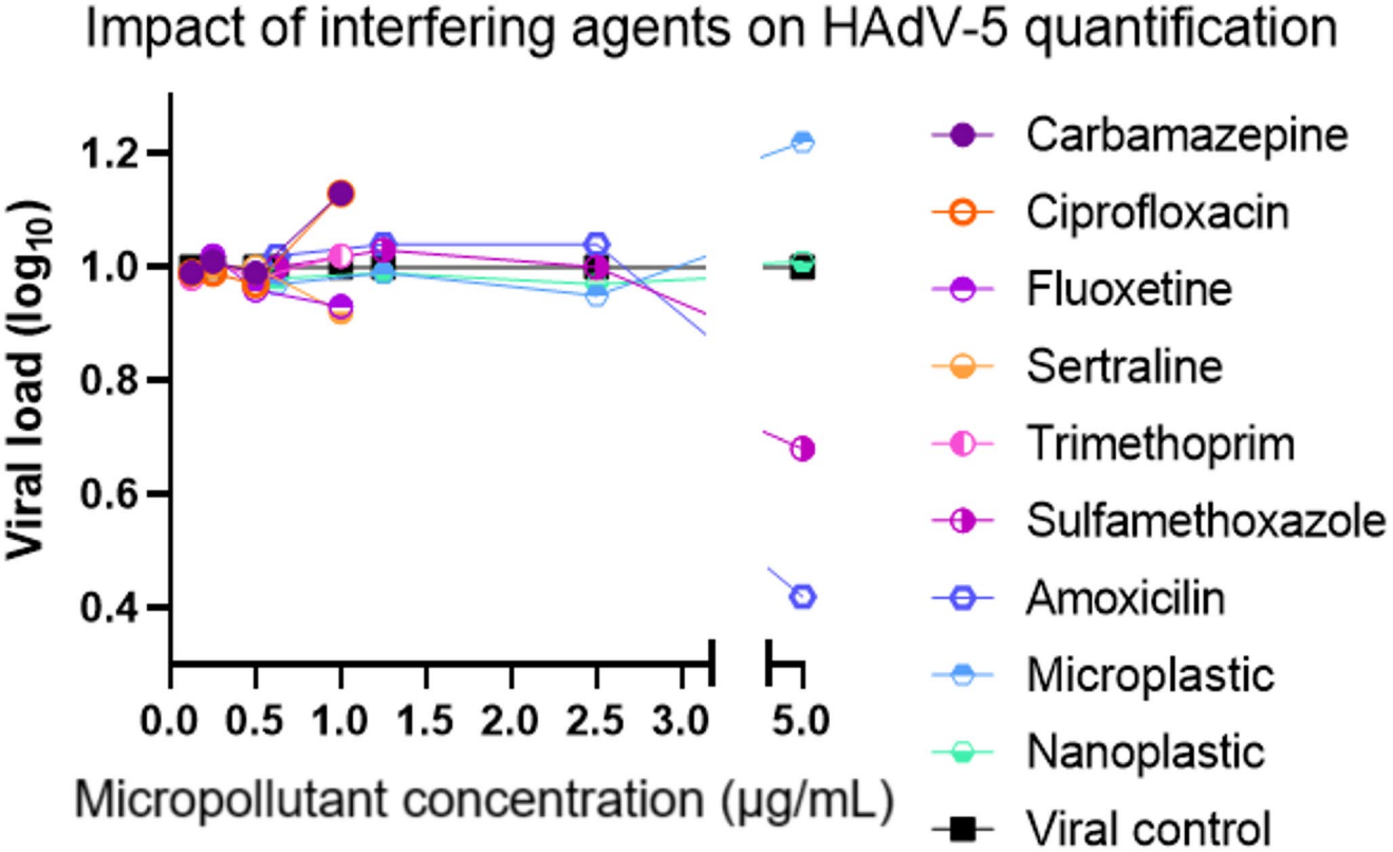
RT-qPCR control to assess potential interactions between micropollutants and the method that could affect quantification of HAdV-5.Reactions were performed in technical duplicates on the RT-qPCR plate, and the values shown follow the same normalization approach used for the in vitro assays, in which the viral control is set to 1 and relative increases or decreases are expressed accordingly

As part of the analytical validation, an additional control assay was carried out to determine whether the micropollutants interfere with the RT-qPCR reaction used for adenovirus quantification (Fig. 2). In this assay, each compound was added directly to samples containing HAdV-5 genetic material, in the absence of cells, to assess potential PCR inhibition or enhancement. Interestingly, at a concentration of 5 µg/mL, both sulfamethoxazole and amoxicillin caused a decrease in detected viral load, suggesting possible inhibition of the RT-qPCR reaction at this concentration. Conversely, polyester microfibers (MPs) induced an increase in detected viral load at the same concentration, which may be related to nonspecific adsorption of viral DNA, matrix effects, or the release of PCR-enhancing substances.

Before selecting the concentrations for the RT-qPCR interference assessment, it is important to note that any PCR-inhibitory effect from the micropollutants would be expected only at levels substantially higher than those initially tested (maximum 5 µg/mL). Moreover, based on the results obtained, interference with HAdV-5 quantification was detected only at concentrations exceeding 5 µg/mL for a subset of micropollutants, and none of these interfering levels approached the concentrations actually used in the experimental assays. It should also be emphasized that the concentrations added to the cell culture do not remain the same throughout the experimental workflow, as the final RT-qPCR input corresponds to the extracted sample, in which the original micropollutant concentrations are considerably diluted. In the in vitro cytotoxicity assays, adverse effects were observed only at concentrations far above 5 µg/mL.

Therefore, the higher, non-cytotoxic concentrations used in the cell culture experiments (Table 3) were selected for subsequent assays because they better approximate the serum levels reported in humans following therapeutic drug administration. These reference values were obtained from literature review, which describe minimum-maximum serum concentrations under standard dosing regimens. This approach was necessary due to the limited availability of studies quantifying bioaccumulation of these compounds in human tissues at levels directly relevant to viral infection models, making serum-based benchmarks a more practical and biologically meaningful basis for experimental design. Furthermore, the concentrations applied in vitro do not directly correspond to intracellular levels, as processes such as uptake, metabolism, and macromolecular binding can significantly alter the bioavailable fraction. Collectively, these considerations highlight the complexity of interpreting in vitro exposure models and support the use of conservative concentrations within physiologically relevant ranges.

### Interactions in Replicative Cycle

**Fig. 3:**
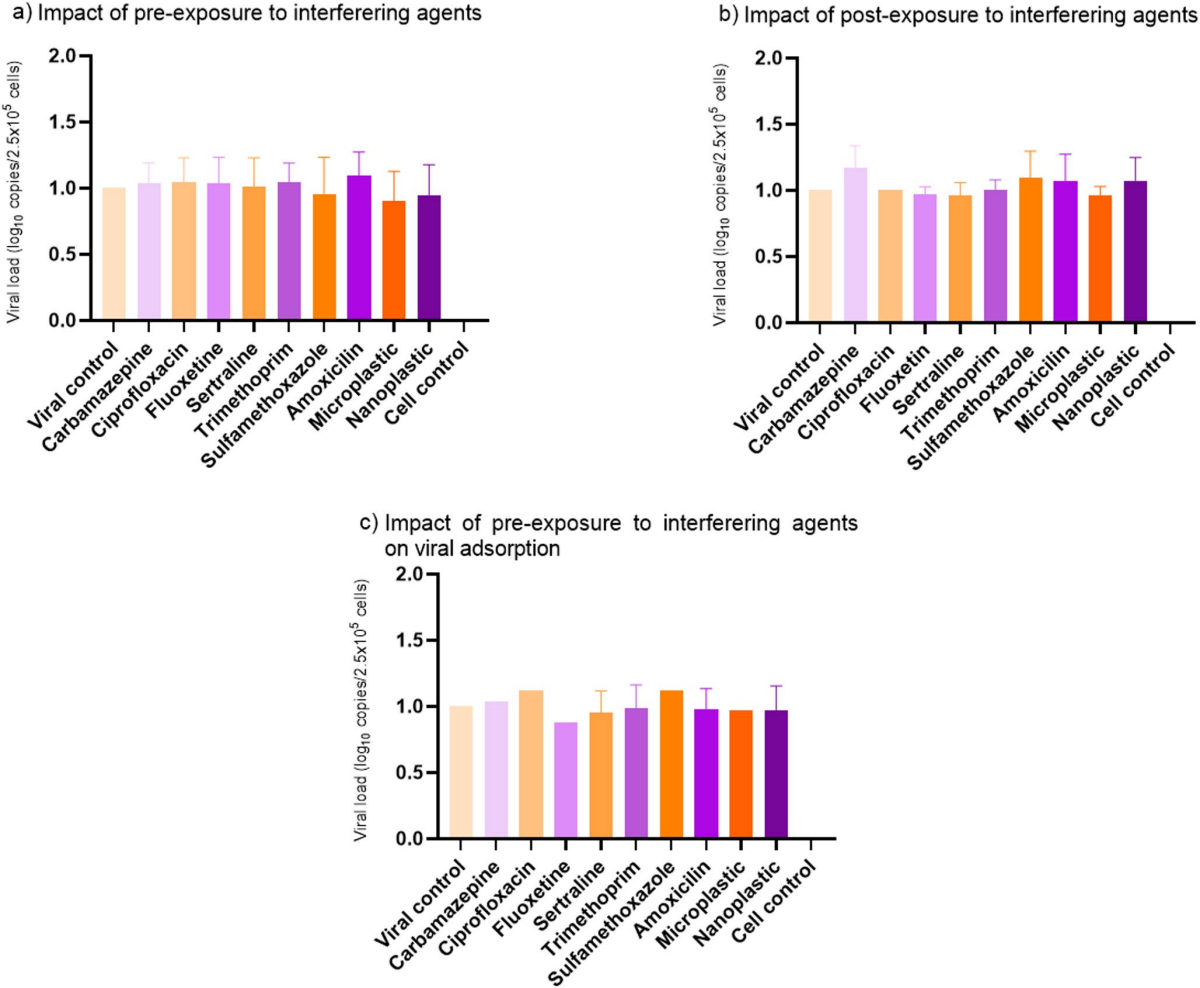
Impact of pre and post-exposure of micropollutants on HAdV-5. Viral control values were normalized to 1 (maximum replication). Values > 1 indicate increased replication relative to the control, and values < 1 indicate reduced replication

The effect of CECs on HAdV-5 replication was evaluated under various exposure protocols simulating different moments of the viral replication cycle. Notably, no significant differences were observed in HAdV-5 mRNA levels when cells were exposed to CECs either prior to infection (cellular pre-exposure, Fig. 3a) nor after infection (post-exposure, Fig. 3b) and during viral adsorption (cellular pre-exposure, Fig. 3c). These findings suggest that the tested CECs, under the concentrations used, do not interfere with cellular susceptibility to viral entry nor with intracellular replication steps such as transcription or assembly. It is likely that the exposure conditions applied did not induce significant physiological changes in the host cells that could impair HAdV-5 replication.

### Simultaneous Exposition Promotes Replication of Adenovirus

**Fig. 4:**
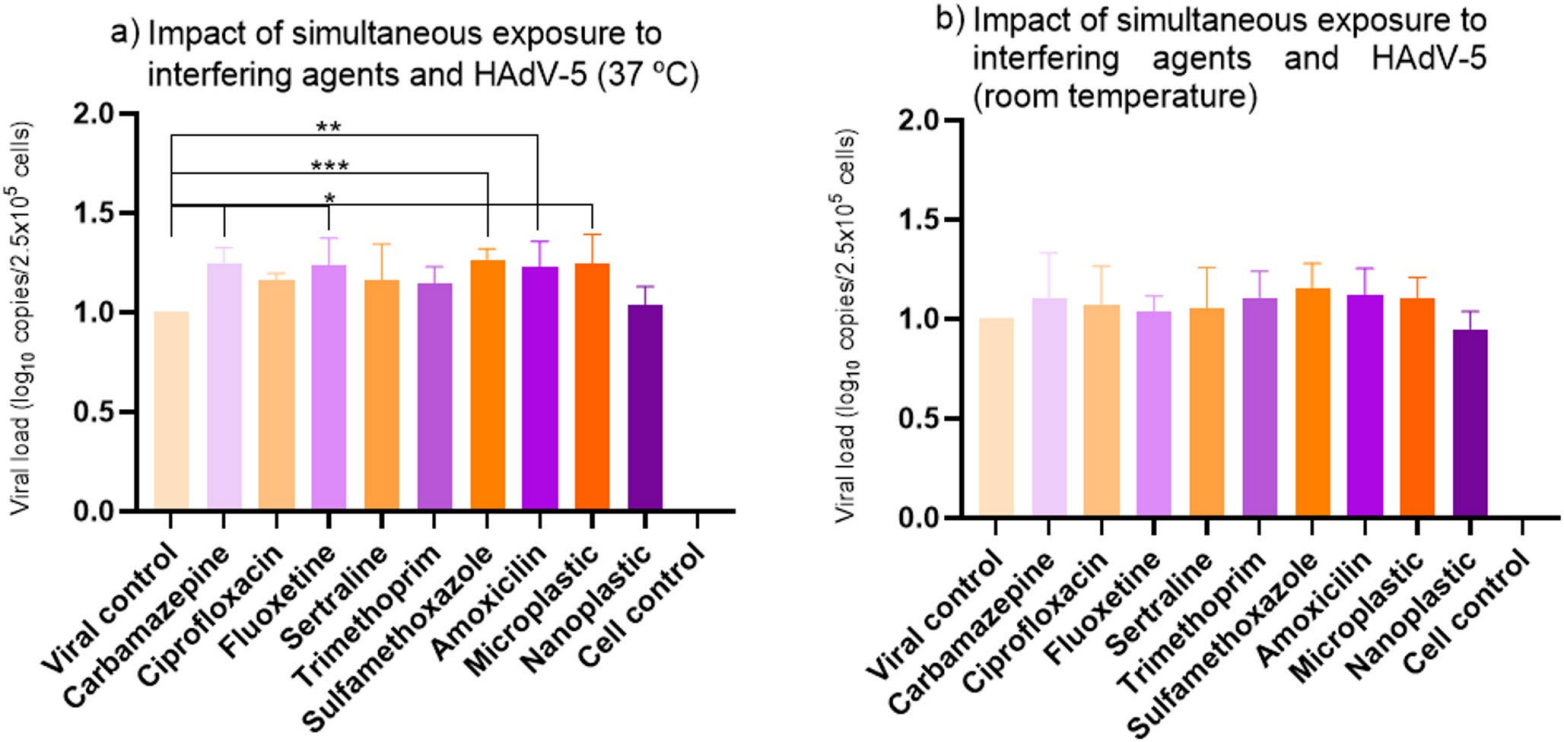
Simultaneous exposure of CECs to HAdV-5 at 37 °C and room temperature. *Carbamazepine, fluoxetine and microplastics *p* < 0.02; **Amoxicillin *p* < 0.03. ***Sulfamethoxazole *p* < 0.008. Viral control values were normalized to 1 (maximum replication). Values > 1 indicate increased replication relative to the control, and values < 1 indicate reduced replication

A significant improvement in viral replication was observed when the co-exposure occurred at 37 °C, indicating that temperature plays a critical role in enhancing interactions between CECs and viral particles, particularly for carbamazepine, fluoxetine, sulfamethoxazole, amoxicillin, and MPs (Fig. 4a). Notably, the enhancement of viral replication under these conditions surpassed 1.5 log₁₀ compared to the viral control, highlighting a biologically relevant increase in viral yield triggered by very short-term interactions. In contrast, no significant changes in viral replication were observed when co-exposure occurred at room temperature (RT) (Fig. 4b).

**Fig. 5:**
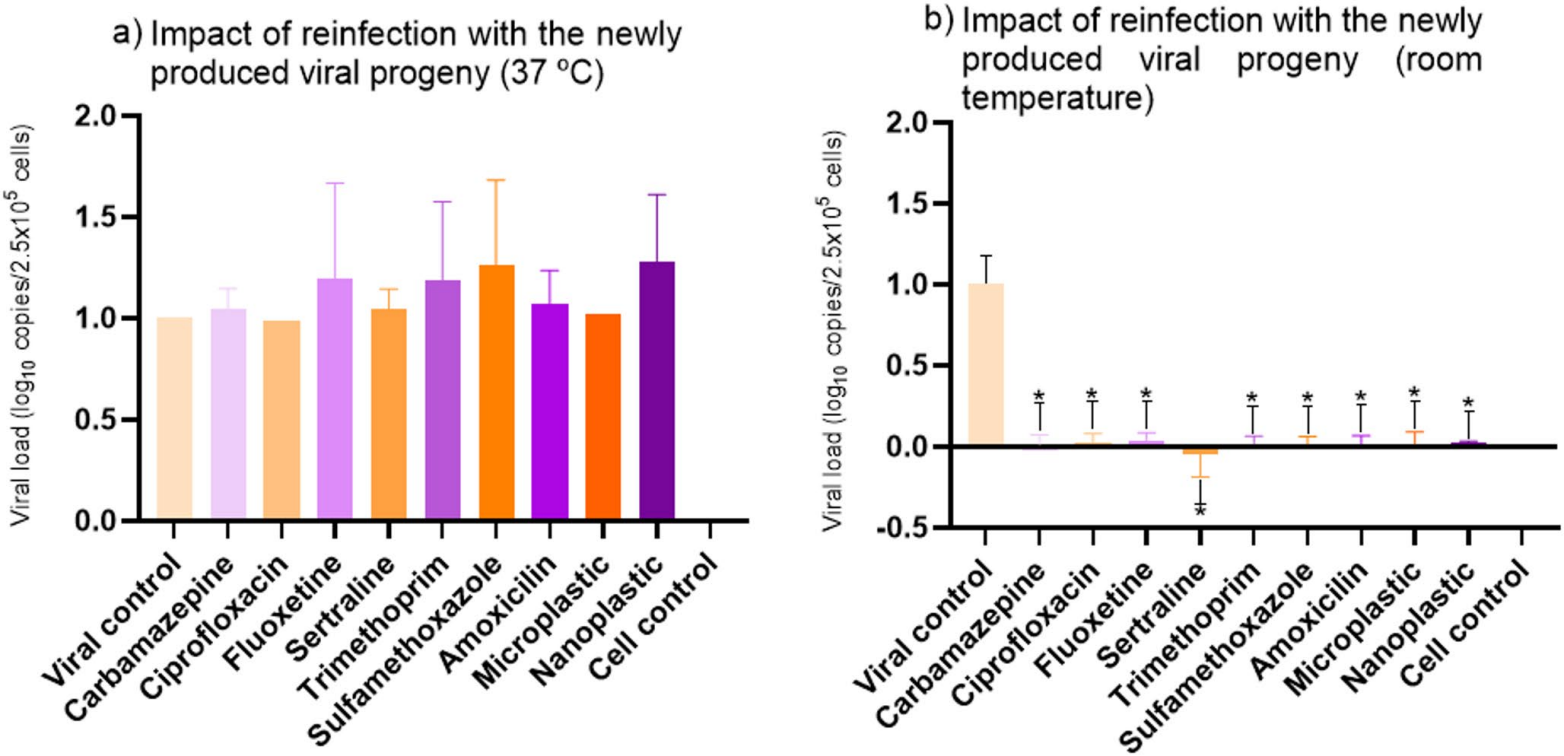
Results from the HAdV-5 newly produced viral progeny at 37 °C and room temperature. Viral control values were normalized to 1 (maximum replication). Values > 1 indicate increased replication relative to the control, and values < 1 indicate reduced replication

In the reinfection assay, supernatants obtained from the 37 °C co-exposure experiment were used to infect fresh A549 monolayers. No significant differences were detected in the replication of the progeny virus compared to the viral control (Fig. 5a). At room temperature, progeny virus levels were significantly lower than the control across all evaluated CECs (Fig. 5b).

## Discussion

The temperature-dependent effects observed in this study are particularly relevant for environmental virology. While room temperature reflects many natural freshwater and surface water conditions, wastewater systems influenced by thermal discharges, seasonal warming, or industrial effluents can reach or approach 30–37 °C (Yan et al., 2023). Under such conditions, interactions between viruses and CECs may be favored, potentially enhancing viral persistence or infectivity in environmentswhere high concentrations of micropollutants are continuously released through human waste. Previous work has shown that microplastics colonized by biofilms can serve as protective microenvironments that reduce viral inactivation rates (Moresco et al., 2022), and that chronic pharmaceutical exposure may influence host-virus interactions (Kong et al., 2021; Previšić et al., 2021), supporting the broader implications of the present findings.

When compared to reinfection at room temperature, HAdV-5 replication was significantly reduced across all micropollutant conditions (Fig. 5b). In the co-exposure assays performed at room temperature, the pronounced decline in viral concentrations in the progeny suggests that micropollutants may inactivate newly produced HAdV-5 particles under environmental conditions. This finding aligns with the possibility that CECs interfere not only with early infection steps but also with late events such as virion assembly, capsid stability, or maturation efficiency.

The temperature-dependent differences further suggest that CEC exposure may influence the quality, not only the quantity, of progeny virions. At lower temperatures, incomplete virion maturation, altered protein processing, or impaired capsid stabilization may render new particles more susceptible to environmental degradation (Sharma et al., 2020). Conversely, at 37 °C the same micropollutants appear to enhance viral replication, indicating that their effects may depend on the physiological state of the host cell and on specific viral morphogenesis pathways that are temperature sensitive.

Another interpretation is that CECs may alter the ratio between infectious and non-infectious particles, as adenovirus infections naturally produce large numbers of defective virions. If micropollutants disrupt late-stage folding or genome packaging, the progeny produced at room temperature may contain a higher proportion of incomplete or defective particles, explaining the reduced reinfection efficiency observed.

Despite the relevance of these findings, several limitations must be acknowledged. First, all experiments were conducted under controlled laboratory conditions, which do not fully capture the complexity of environmental systems or human physiology. An important limitation of this study is the uncertainty regarding the exact chemical or physical form responsible for the observed effects. At physiological temperature, increased molecular motion could facilitate binding, adsorption, or even partial encapsulation of viral particles by CECs, impairing their ability to interact with cellular receptors. Moreover, in real biological systems, pharmaceuticals undergo extensive metabolism, resulting in a variety of active or inactive metabolites that may differ significantly in their physicochemical properties and biological activity (Yan et al., 2023). In this context, it remains unclear whether the effects observed in vitro are primarily driven by the parent compound or by its metabolites, which may be present in higher concentrations in excreta and environmental matrices (Han & Lee, 2017). Future studies should aim to differentiate between the direct impact of the original pharmaceutical molecule and that of its biotransformation products, especially considering that some metabolites may retain, enhance, or even reverse the biological effects of the parent drug. This distinction is critical to improve the environmental relevance of experimental models and to more accurately predict the fate and influence of pharmaceutical residues in natural and engineered ecosystems.

The interaction between viruses and micropollutants, particularly MPs, is an emerging field with important implications for environmental virology. As highlighted by Yang et al. (2023), MPs can serve as carriers or scaffolds for various microorganisms, including viruses, potentially altering their environmental persistence, modes of host entry, and interactions with immune defenses. This biotic–abiotic interface can influence not only viral stability but also the cellular mechanisms of viral uptake and replication. In the present study, we observed changes in adenovirus replication when co-exposed to micropollutants during cell culture assays, supporting the hypothesis that chemical contaminants may modulate viral dynamics. Although the exact mechanisms of such interactions remain unclear, our findings reinforce the relevance of considering the combined effects of physical particles and chemical pollutants on viral infectivity and replication. These insights emphasize the need for integrative approaches in risk assessment of contaminated matrices, especially in the context of waterborne viruses and environmental health.

Recent studies have demonstrated that MPs can enhance viral infection and facilitate the adsorption of viruses onto MPs (Lu et al., 2022). Wang et al. (2023) used confocal fluorescence microscopy to reveal co-localization between polystyrene PS particles and the nucleoprotein of Influenza A Virus (IAV), indicating that PS particles act as carriers, enriching viral particles and promoting their uptake via endocytosis. Beyond facilitating entry, PS exposure suppressed critical antiviral mechanisms, including the RIG-I-like receptor (RLR) signaling pathway, by inhibiting the phosphorylation of TBK1 and IRF3, downregulating the expression of IFN-β, and significantly reducing the levels of IFITM3, a key protein that restricts viral membrane fusion and replication. In the present study, although focused on human adenovirus, we similarly observed alterations in viral replication patterns following exposure to environmental micropollutants in cell culture. These findings suggest that, as with IAV, chemical contaminants may influence not only the entry but also the replication efficiency of viruses, either directly or through modulation of host cell pathways. While the exact molecular mechanisms remain to be elucidated for adenovirus, our results reinforce the hypothesis that micropollutants may compromise cellular defenses and promote viral replication, highlighting the importance of further investigating these interactions across different viral models and environmental contaminants.

The findings by Moresco et al. (2022) demonstrate that MPs colonized by biofilm constitute a favorable microenvironment for viral adsorption and protection, reducing viral inactivation rates and thereby enhancing stability, persistence, and potential dissemination of infectious viruses, both enveloped and non-enveloped, in aquatic ecosystems. Complementarily, our in vitro study shows that co-exposure to micropollutants, including MPs, under physiological temperature conditions (37 °C), markedly enhances viral replication, indicating a synergistic modulation of viral infectivity by chemical and physical factors. This perspective is further substantiated by the recent review by Shruti et al. (2024), who highlight emerging research demonstrating that plastispheres influence viral population dynamics, diversity, and functions, and that viral interactions with MPs may alter host-associated environments. The authors frame these findings within an evolving paradigm wherein plastispheres are viewed as dynamic microhabitats with the potential to shape viral ecology, drive evolutionary processes, and impact public health. Collectively, this synthesis underscores the urgent need for integrative mechanistic studies to elucidate how environmental factors, such as temperature, chemical co-contaminants, and the structural complexity of plastisphere communities, interact to modulate viral persistence, infectivity, and dissemination in contaminated ecosystems.

Together, these findings highlight the importance of exposure timing and environmental conditions (notably temperature and time of exposition) in shaping the interaction between micropollutants and viral particles.

## Conclusion

In conclusion, our findings demonstrate that exposure to selected CECs significantly enhances HAdV-5 replication in vitro, indicating that micropollutants can directly modulate viral infectivity under physiologically relevant conditions. When considered together with environmental evidence showing that microplastics and their biofilms can protect viruses and prolong their persistence in aquatic systems (Moresco et al., 2022), as well as recent insights that plastispheres may influence viral ecology (Shruti et al., 2024), our results reinforce the emerging view that contaminated environments may actively shape viral behavior. These observations highlight an important and previously underrecognized dimension of viral–pollutant interactions. Overall, this study emphasizes the need to incorporate chemical and physical co-contaminants into future viral risk assessments, and points to the importance of mechanistic investigations to fully understand how environmental micropollutants affect viral persistence, infectivity, and potential dissemination.

## Data Availability

Data will be made available on request.
